# Quality of life in chemical warfare survivors with ophthalmologic injuries: the first results form Iran Chemical Warfare Victims Health Assessment Study

**DOI:** 10.1186/1477-7525-7-2

**Published:** 2009-01-19

**Authors:** Batool Mousavi, Mohammad Reza Soroush, Ali Montazeri

**Affiliations:** 1 Janbazan Medical and Engineering Research Center (JMERC), Tehran, Iran; 2 Iranian Institute for Health Sciences Research, ACECR, Tehran, Iran

## Abstract

**Background:**

Iraq used chemical weapons extensively against the Iranians during the Iran-Iraq war (1980–1988). The aim of this study was to assess the health related quality of life (HRQOL) in people who had ophthalmologic complications due to the sulfur mustard gas exposure during the war.

**Methods:**

The Veterans and Martyrs Affair Foundation (VMAF) database indicated that there were 196 patients with severe ophthalmologic complications due to chemical weapons exposure. Of these, those who gave consent (n = 147) entered into the study. Quality of life was measured using the 36-item Short Form Health Survey (SF-36) and scores were compared to those of the general public. In addition logistic regression analysis was performed to indicate variables that contribute to physical and mental health related quality of life.

**Results:**

The mean age of the patients was 44.8 (SD = 8.7) ranging from 21 to 75 years. About one-third of the cases (n= 50) reported exposure to chemical weapons more than once. The mean exposure duration to sulfur mustard gas was 21.6 years (SD = 1.2). The lowest scores on the SF-36 subscales were found to be: the role physical and the general health. Quality of life in chemical warfare victims who had ophthalmologic problems was significantly lower than the general public (P < 0.001). The results obtained from logistic regression analysis indicated that those who did not participate in sport activities suffer from a poorer physical health (OR = 2.93, 95% CI = 1.36 to 6.30, P = 0.006). The analysis also showed that poor mental health was associated with longer time since exposure (OR = 1.58, 95% CI = 1.04 to 2.39, P = 0.03) and lower education (OR = 3.03, 95% CI = 1.21 to 7.56, P = 0.01).

**Conclusion:**

The study findings suggest that chemical warfare victims with ophthalmologic complications suffer from poor health related quality of life. It seems that the need for provision of health and support for this population is urgent. In addition, further research is necessary to measure health related quality of life in victims with different types of disabilities in order to support and enhance quality of life among this population.

## Background

During the 1980–1988 Iran-Iraq war, the human cost to Iran included more than 200,000 lives lost and more than 400,000 of persons injured, of whom more than 50,000 were exposed to chemical warfare agents especially sulfur mustard gas [1]. Sulfur mustard gas is an alkylating agent that has serious, toxic effects on skin, eyes and respiratory system [2].

War has a far-reaching impact on the health and well being of the soldiers, war veterans, and victims and even on the population as a whole [3]. The impact of war on soldiers', and veterans' health has been widely studied [3-6]. Veterans not only suffer from worse health conditions than non-veterans [3,5-8], but they also have a greater illness burden, and higher mortality rates resulting in a substantial increase in their use of health care facilities [3,7,9].

Health related quality of life (HRQOL) has been measured in various groups of veterans in different settings [3-8,10-16], but little is known about chemical warfare victims' health related quality of life. Chemical warfare victims face different types of complications and disabilities due to sulfur mustard gas exposure. Thus, as mentioned earlier, since in Iran there are about 50,000 chemical warfare victims both among veterans and the general public it was decided to conduct a study to examine victims' health status in order to meet their needs. The study is known as Iran Chemical Warfare Victims Health Assessment Study, and includes examinations of all complications due to chemical warfare agents among veterans and civilians. This is the first part of the study that assesses health related quality of life in chemical warfare victims who developed severe ophthalmologic problems. It has been shown that severely intoxicated ophthalmologic patients present with delayed keratitis, corneal vascularization, thinning, and epithelial defect [17]. Thus, since eyes are a very sensitive human organs and have tangible effects on individuals' every day life, vision-related quality of life is an important area that needs to be understood further [18,19]. To our best knowledge this is the first study that reports on the topic.

## Methods

### Design and data collection

All injured survivors (both civilians and veterans) of the Iran-Iraq war are given a severity index (disability rate) in the Veterans and Martyrs Affair Foundation (VMAF) database, based on their clinical problems and severity of the injury or injuries. Since the Foundation provides special services and complementary facilities for injured survivors, it is believed that most injured are registered with the Foundation. In other word without registration injured survivors could not get the services that they are needed. Thus, the VMAF database keeps all the victims' (n = 50,000) demographic and clinical information. Most common complications recorded in the database are lungs (42.5%), eyes (39.3%), and skin (24.5%) related complications. Of these only a small proportions (0.023 to 1%) of injured are considered having severe complications [20]. We extracted the data for all cases that had severe ophthalmologic complications due to exposure to sulfur mustard gas agent during the 8 years of the Iran-Iraq war. According to medical documents in the VMAF database 196 patients had severe ophthalmologic complications. The patients were from 21 provinces of Iran. One hundred forty-seven (n = 147) patients gave informed consent to participate in the study. In order to collect data, semi structured interviews were conducted by 3 trained assessors. Each patient was interviewed separately, face-to-face, for about 15–20 minutes. The remaining patients (n = 49) did not participate in the study due to dislike. A team of trained interviewers collected data and all participants were interviewed in their home.

Data for a general Iranian population derived from a population-based study of a random sample of the 4163 individuals aged 15 years and over living in Tehran, Iran. To select a representative sample of the general population the study used a stratified multi-stage area sampling approach. Every household within 22 different districts in Tehran had the same probability to be sampled and given that Tehran has became a multicultural metropolitan area it has been suggested that a sample from the general population in Tehran at least could be regarded as a representative sample of urban population in Iran. In addition since all the study participants in the current investigation were male, we used information for males only, that is the comparison was made between the patients' scores on the SF-36 with those of the male genders from the general population [21].

### Quality of life measure

Quality of life was measured using the 36-item Short Form Health Survey (SF-36). The SF-36 is a generic tool that can be used for the general population and different patients groups. The questionnaire consists of 36 questions that measure eight health-related concept. It also provides two summary scales: Physical Component Summary (PCS) and Mental Component Summary (MCS). Scores on each of the subscales range from 0 to 100, with 0 representing the worst health-related quality of life and 100 representing the best [22].

The psychometric properties of the Iranian version of the SF-36 were examined in a previous study and it has been shown that the internal consistency (to test reliability) for all eight SF-36 scales met the minimum reliability standard, the Cronbach's a coefficients ranging from 0.77 to 0.90 with the exception of the vitality scale (alpha = 0.65). Known groups comparison showed that in all scales the SF-36 discriminated between men and women, and old and the young respondents as anticipated (all p values less than 0.05). Convergent validity (to test scaling assumptions) using each item correlation with its hypothesized scale showed satisfactory results (all correlation above 0.40 ranging from 0.58 to 0.95). Factor analysis identified two principal components that jointly accounted for 65.9% of the variance [21].

### Additional information

Demographic data were collected with regard to age, sex, level of education, marital status, and employment status for the victims. Additionally, data were collected on time and frequency of chemical agent exposure, other war-related injuries and psychological problems, and history of hospitalization during the last year.

### Statistical analysis

In addition to descriptive statistics, the patients' scores on the SF-36 were compared with those of a general Iranian population using one sample t-test and independent t-test.

We performed logistic regression analysis to determine variables that most contribute to health-related quality of life in patients with severe ophthalmologic complications due to exposure to sulfur mustard gas agent. For the purpose of the logistic regression analysis Physical Component Summary (PCS) and Mental Component Summary (MCS) were used as dependent variables and age, education, employment status, frequency of chemical exposure, time since last exposure, other war related injuries, history of hospitalization and sport activities considered as independent variables. Relative to the mean PCS and MCS scores the study sample was divided into two groups, those who scored equal or greater than mean (PCS: n = 64; MCS: 63) and those who scored below mean (PCS: n = 83; MCS: n = 84). As a rough guide the mean score for any given population seems to be the best cut-off point to determine whether a group or individual scores above or below the average [23].

### Ethics

The Ethics Committee of Janbazan Medical and Engineering Research Center (JMERC), Tehran, Iran approved the study. All patients gave consent.

## Results

### Patients' characteristics

The relevant socio-demographic and clinical characteristics of the victims (n = 147) are shown in Table 1.

**Table 1:** Demographic characteristics of Iranian chemical warfare survivors with ophthalmologic injuries (n= 147)

	**Number**	**Percentage**
**Age**		

<30	13	8.8

30–39	36	24.5

40–49	80	54.4

>50	18	12.3

Mean (SD)	44.8 (8.6)	

**Marital status**		

Married	144	98

Single	3	2

**Education (years)**		

0–8	42	28.6

9–12	63	42.9

> 12	42	28.6

**Employment status**		

Employed	37	25.2

Unemployed	110	71.4

**History of hospitalization**		

No	41	27.9

Yes	106	72.1

**Extent of eye injury**		

One eye	71	48.3

Two eyes	76	51.7

**Frequency of chemical agent exposure**		

1 exposure	98	66.3

>1 exposure	49	33.7

**Mean time since exposure (SD)**	21.6 (1.2)	

**Age at exposure**		

< 20	55	37.4

≥ 20	92	62.6

Mean (SD)	23.2 (8.4)	

**Sport activities**		

No	67	45.6

Yes	80	54.4

Other war related Injuries and psychological problems (n = 73)*		

Injury of extremities	42	57.3

Psychological problems	24	32.9

Head injuries	20	27.4

Face injuries	12	16.4

* Some patients reported more than one war related injuries.

### Quality of life

1. Comparison of the SF-36 scores between patients and the general population: the mean scores of chemical warfare victims on the SF-36 were significantly lower than the general Iranian population on all measures (Table 2).

**Table 2:** Comparison of the SF-36 scores between chemical warfare patients and a general Iranian population (higher scores indicate a better condition)

	**Patients (n = 147)**	General population (n = 1997)*	
	**Mean (SD)**	**Mean (SD)**	**P**

**Physical functioning**	45.3 (19.5)	87.8 (19.0)	< 0.0001

**Role physical**	23.1 (16.6)	73.8 (36.4)	< 0.0001

**Bodily pain**	29.5 (16.8)	82.7 (23.4)	< 0.0001

**General health**	26.3 (14.8)	70.2 (19.6)	< 0.0001

**Vitality**	39.7 (18.8)	68.9 (16.2)	< 0.0001

**Social functioning**	47.6 (21.6)	78.0 (23.5)	< 0.0001

**Role emotional**	43.5 (26.6)	70.1 (39.7)	< 0.0001

**Mental health**	47.8 (21.2)	69.2 (17.1)	< 0.0001

**Physical Component Summary (PCS)**	27. 4 (14.8)	81.4 (21.8)	< 0.0001

**Mental Component Summary (MCS)**	39.9 (19.4)	72.4 (21.9)	< 0.0001

* Derived from [22]. The scores are for males only.

2. Results obtained from logistic regression analysis: in order to find out predicting factors for poor physical and mental health related quality of life, the logistic regression analysis was performed and the results indicated that those who did not participate in sport activities suffer from a poorer physical health (OR = 2.93, 95% CI = 1.36 to 6.30, P = 0.006). The analysis also showed that poor mental health was associated with longer time since exposure (OR = 1.58, 95% CI = 1.04 to 2.39, P = 0.03) and lower education (OR = 3.03, 95% CI = 1.21 to 7.56, P = 0.01). For both PCS and MCS the other variables that entered into the regression models did not show significant results, although higher risks were observed in the expected directions. The results are shown in Table 3.

**Table 3:** Determinants of poor physical and mental health related quality of life in Iranian chemical warfare survivors with ophthalmologic injuries (n = 147)

	**OR (95% CI)**	**P**
**Physical Component Summary (PCS)**		

*Age*	1.03 (0.97–1.08)	0.29

*Education (years)*		

> 12	1.0 (ref.)	

9–12	2.17 (0.91–5.22)	0.08

0–8	1.89 (0.61–5.88)	0.27

*Employment status*		

Employed	1.0 (ref.)	

Unemployed	1.54 (0.64–3.72)	0.32

*Frequency of chemical exposure*		

Once	1.0 (ref)	

More than once	1.07 (0.46–2.47)	0.86

*Time since last exposure*	0.94 (0.68–1.32)	0.75

*Other war related injuries*		

No	1.0 (ref.)	

Yes	1.12 (0.49–2.52)	0.77

*History of Hospitalization*		

No	1.0 (ref.)	

Yes	1.04 (0.45–2.40)	0.92

*Sport activities*		

Yes	1.0 (ref.)	

No	2.93 (1.36–6.30)	0.006

**Mental Component Summary (MCS)**		

*Age*	1.02 (0.97–1.07)	0.36

*Education (years)*		

> 12	1.0 (ref.)	

9–12	3.03 (1.21–7.56)	0.01

0–8	1.84 (0.57–5.88)	0.30

*Employment status*		

Employed	1.0 (ref.)	

Unemployed	1.27 (0.51–3.14)	0.59

*Frequency of chemical exposure*		

Once	1.0 (ref.)	

More than once	1.44 (0.62–3.37)	0.39

*Time since last exposure*	1.58 (1.04–2.39)	0.03

*Other war related injuries*		

No	1.0 (ref.)	

Yes	1.61 (0.71–3.68)	0.25

*History of Hospitalization*		

No	1.0 (ref.)	

Yes	1.79 (0.75–4.25)	0.18

*Sport activities*		

Yes	1.0 (ref.)	

No	1.97 (0.89–4.35)	0.09

## Discussion

Although a number of limited studies measured quality of life in survivors of the Iran-Iraq war [14-16], the present study is the first survey of quality of life in Iranian chemical warfare survivors. The findings of the present study revealed that patients suffer from poor quality of life. They scored very low on the SF-36 compared to both existing national and international data [4,5,7,21,24-26]. The findings indicated that patients particularly scored lower on the role physical and general health subscales. This perhaps is an indication that patients need more support from the healthcare system.

In general victims scored better on mental health related subscales than physical health dimensions (Table 2). This might be explained by two general impressions usually one can observe among Iran-Iraq war victims. First, since most Iranian war victims were the volunteer veterans and civilians thus they coped better with their conditions. Secondly, they enjoy from having a supportive family environment. Further investigations of relationship between victims' mental health and these factors are recommended.

Sport activity was the only significant contributing variable to the physical component summary score (Table 3). Physical component summary (PMC) provides a relatively objective indicator of physical health because it describes the physical ability, limitations and difficulties in performing everyday duties and cutting down the amount of time spent on activities. Differences between subgroups of patients who differed in sport activities could be due to the fact that perhaps the above variable had significant impact on physical functioning as well as role physical [4,19-26]. Thus, those who did not perform sport activities showed a significant poorer physical health related quality of life compared to those who did perform physical activities.

There were a significant association between level of education (9 to 12 years education category), and time since exposure and mental component summary score (MCS). The association between low education and poor mental health might be due to the fact that the SF-36 is highly dependent on education. In addition the association might be a reflection of association between low education and high risk for traumatization. For instance, it is argued that the risk for developing post-traumatic stress disorder (PSTD) depends on several factors including pre-military educational attainment [27]. However, the significant contribution of time since exposure indicates that as time passes the risk for poorer mental health related quality of life is increasing (OR = 1.58). This suggests that healthcare system should be more concerned about older victims and provide necessary supportive interventions for this group of patients. It has been shown that age play important role in increased PSTD [28].

We did not observed significant results for association between poor physical and mental health and other war related injuries and psychological problems. It might argue that since exposure to mustard gas had serious impacts on the victims' health status, so additional accompanying war traumas did not make a significant contribution to their present physical and mental health related quality of life. It seems that there is need to carry out additional investigations using more cases to shade more light on the topic.

There were no significant association between poor physical and mental health related quality of life and independent variables such as age, employment status, frequency of chemical exposure, and history of hospitalization. However, in all instances the analysis showed higher risks of poor physical and mental health summary scores and these factors in the expected directions (Table 3).

This study has several limitations. The survey was a descriptive study in nature and therefore does not imply causation. In addition, since one-third of the eligible chemical warfare survivors with ophthalmologic complications did not participate in the study, the patients in the sample might not be completely representative of this population and thus the results might not be generalized.

## Conclusion

The results strongly suggest that chemical warfare survivors with ophthalmologic complications suffer from poor health related quality of life. The findings imply that healthcare system should provide supportive strategies and interventions appropriate to the situation of this population. In addition, further research is necessary to measure health related quality of life in victims with different types of disabilities in order to support and enhance quality of life among this population.

## Abbreviations

HRQOL: Health-related quality of life; VMAF: Veterans and Martyrs Affair Foundation; PF: Physical functioning; RP: Role physical; BP: Bodily pain; GH: General health; VT: Vitality; SF: Social functioning; RE: Role emotional; MH: Mental health

## Competing interests

The authors declare that they have no competing interests.

## Authors' contributions

BM was the principal investigator and was responsible for the study design, data analysis, and wrote the first draft. MRS and BM collected the SF-36 data and extracted patients' case records. AM analyzed the data and wrote the final manuscript. MRS, AM and BM actively contributed to all elements of the study. All authors read and approved the final manuscript.
